# Classifying Female Sexual Homicide Offenders: A Latent Class Analysis of Murder Arrestees in the U.S.

**DOI:** 10.1002/bsl.2709

**Published:** 2024-12-08

**Authors:** Heng Choon (Oliver) Chan, Matt DeLisi, Timothy G. Edgemon

**Affiliations:** ^1^ Department of Social Policy, Sociology, and Criminology School of Social Policy and Society University of Birmingham Birmingham UK; ^2^ Department of Sociology and Criminal Justice Iowa State University Ames Iowa USA

**Keywords:** characteristics, female offender, latent class analysis, sexual homicide, sexual homicide offender, sexual murder, sexual murderer, typology

## Abstract

Research on sexual homicides has primarily focused on male offenders, and thus little is known about female offenders who perpetrated sexual homicides. This study aimed to develop the first statistical classification of female single‐victim (SV) sexual homicide offenders (SHOs) using the U.S. FBI's Supplementary Homicide Reports database that spanned over a 47‐year period (1976–2022). A latent class analysis (LCA) was computed to detect subtypes of female SHOs in a sample of 158 offenders. Findings of the LCA identified two unique classes of female SHOs exist within the data: White intra‐familial offenders and Black extra‐familial offenders. The distinguishing features of these two classes were the offender's racial group, the victim's age and racial groups, the offender‐victim relationship, the geographical urbanness level of crime location, and weapon use. This empirically‐derived offender classification can be informative to law enforcement agents and security professionals in their investigative strategies.

## 1 Introduction

Sexual victimization is both a violation of human rights and a public health concern globally that occurs in all ages, sexes, ethnicities, educational fields, and socioeconomic groups. Sexual offending, in general, is committed under different circumstances and contexts, such as illegal sexual penetration (i.e., rape) and unwanted sexual contact (e.g., fondling). In severe instances, sexual assault can lead to the death of the victim—that is, sexual homicide. Although sexual homicides garner headlines and dominate the news globally, the occurrence of these criminal events is rare. While the prevalence of sexual homicides varies across countries, the estimates have been reported to be 1%–5% of all homicides (Chan 2019). These countries include the U.S. (0.84%; Chan 2021), Australia (0.9%; Mouzos 2003), Canada (2.7%; Kong 2003), Finland (2.8%; Häkkänen‐Nyholm et al. 2009), England and Wales (3.7%; Francis and Soothill 2000), and Jamaica (5%; Lemard and Hemenway 2006).

Although there is a growth in academic interest on the study of sexual homicides, only 32 empirical studies were published in a 32‐year period between 1986 and 2007 (Chan and Heide 2009), with this increasing to 47 empirical studies published in the subsequent eight years (2008–2015; Chan 2017). It should be noted that over 97% of these empirical studies examined male sexual homicide offenders (SHOs) and their offending patterns. To our knowledge, only a handful of studies to date have empirically examined the *modus operandi* (MO; also known as methods of operation) of female sexual murders. More specifically, only five empirical studies published on sexual homicides involved female offenders, with four studies on American female SHOs (Chan 2021; Chan and Frei 2013; Chan, Frei, and Myers 2013 2019) and another on Scottish female sexual murderers (Skott, Beauregard, and Darjee 2019). Hence, there is more to be learned about this under‐researched offender population. The current study is significant and unique in filling a gap in knowledge about female sexual homicide offending.

### 1.1 The Prevalence Rate of Female‐Perpetrated Sexual Offenses and Homicides

Research on female‐perpetrated sexual offending is limited at best. While the phenomenon of sexual offending by females is becoming more widely recognized empirically, it is critically under‐researched in comparison to sexual offending by males (Chan 2023; Gannon and Rose 2008). The prevalence estimates of female sex offenders vary across different countries with about 5%–6% of all sex offenders in the U.S., Canada, the U.K., New Zealand, and Australia (Cortoni and Hanson 2005). Although official reports often documented a lower prevalence rate, self‐report studies, in contrast, have suggested that the actual rate of perpetration was much higher. For instance, Devon (2003) reported that 5% of male victims of sexual abuse and 20% of female victims identify a woman as the offender; while Schwartz and Cellini (1995) found that approximately 63% of female victims and 27% of male victims identified their perpetrator as a woman. It has been suggested that sexual victimization by females often goes undetected because these acts are normally committed during caretaking activities where such behaviors were unlikely to be suspected as sexually deviant, especially when the victims were children (Ferguson and Meehan 2005; Groth 1979). Nevertheless, it is utterly important to recognize that women are also equally capable of engaging in sexual aggressive behavior (Chan 2023).

Similarly, research on female‐perpetrated homicides has always been scarce primarily due to the low murder arrest rate of female offenders. According to the U.S. Federal Bureau of Investigation's (FBI) Uniform Crime Report (UCR), female offenders constituted 10.6% of the overall arrested murderers in 2022 (FBI 2023). This figure is relatively stable over the past five years (2017–2021) with a range from 8.8% to 11.5% annually (FBI, n.d.). Similar prevalence estimates of female murderers were also reported in European countries (e.g., Finland, the Netherlands, Sweden, and England and Wales) with an average rate of 10% of all reported homicides (Flynn et al. 2011; Liem et al. 2013). Many of these female‐perpetrated murders were domestic in nature, with their killings were generally claimed to be unplanned actions or acted in self‐defense (Goetting 1995; Smith, Basile, and Karch 2011). Despite this, the low arrest rate made research on female homicide offenders a challenging task.

### 1.2 Female Sexual Homicides and Sexual Murderers

Similar to sexual offenses and homicides in general, a large majority of sexual homicides are committed by male offenders (95%; Chan, Myers, and Heide 2010), with less than 5% of those arrested for this violent offense identified as females (Myers and Chan 2012). Given the low prevalence rate, only a handful of empirical studies have examined the offending dynamics of sexual homicides perpetrated by female offenders. The work by Chan and Frei (2013) was arguably the first empirical study of female sexual murderers. Analyzing 204 sexual homicides committed by female offenders over a period of 32 years (1976–2007) extracted from the FBI's Supplementary Homicide Reports (SHRs), they found that White (53%) and Black (47%) were somewhat equally distributed in the U.S. female SHO population. These female offenders were more likely to target adult males as victims and their homicide was intra‐racial in nature (i.e., White‐against‐White and Black‐against‐Black). This study also found that female SHOs primarily used firearms in killing their victims, especially against adult male victims who presumably were physically stronger than them.

In their follow‐up study, Chan, Frei, and Myers (2013) further examined the racial differences and similarities in female sexual homicide offending patterns of 204 female SHOs (105 Whites and 94 Blacks). The findings indicated that most female SHOs, regardless of race, murdered victims of the opposite sex (i.e., heterosexual murders). Female SHOs of both races (44% of Whites and 57% of Blacks) were more likely to target known victims (e.g., friends and acquaintances) who were not intimate partners or family members. Although firearms remain as the most commonly used murder weapons by female SHOs from both races (60% of Whites and 48% of Blacks), edged weapons (32%) were the next frequently used weapon by Black offenders, while personal weapons (17%) were next favored by White offenders. In terms of the crime scene location, Black female SHOs were more likely to perpetrate their offense in large cities (69%), whereas White female SHOs preferred to commit their murder in suburban areas (40%).

The next empirical study that involved female SHOs was conducted by Chan, Heide, and Beauregard (2019) on sex differences in the types of murder weapons used by 3009 male and 151 female sexual murderers in the U.S. analyzing a 37‐year period (1976–2012) of SHR dataset. Findings indicated that female SHOs were more likely to use firearms (63%), while male SHOs more preferred to use personal weapons (43%) in killing their victims. It is important to note that female sexual murderers predominantly used weapons that were physically less demanding (e.g., firearms, and edged and other weapons; 89%) in their offense commission.

In addition to Chan et al.’s studies of female SHOs using the SHR dataset, Skott, Beauregard, and Darjee (2019) was the only other study that have examined the offending characteristics of female sexual murderers. In addition to 106 cases of female nonsexual homicides and 89 case of male sexual homicides, they identified seven cases of female sexual homicides in their analysis of a 26‐year period (1990–2015) of the Scottish Homicide Database. Skott, Beauregard, and Darjee (2019) found that female sexual murderers were most likely Whites (100%), were proportionally often unemployed (100%), were unlikely to be homeless (100%), and were younger than their victims (the most common offender age group was 31–45 years compared to the most common victim age group was 46 years or older). They were also more likely to engage in intra‐racial (i.e., Whites murdered Whites; all seven cases) and heterogenous (i.e., females murdered males; 4/7 cases) sexual homicides. Relative to male sexual homicides, female SHOs were more likely to commit their murder with an accomplice (71%) and to attack a family member (14%). Compared to female nonsexual homicide offenders and male SHOs, female sexual murderers were more likely to use physical assault when killing their victims (57%) and the crime scene to be an indoor location (86%).

More recently, Chan (2021) conducted a study comparing 243 sex worker homicides (189 male and 54 female offenders) with 2608 sexual homicides (2474 male and 134 female offenders). Analyzing the single‐victim, single‐offender homicide cases extracted from a 37‐year (1976–2012) SHR database, Chan found that there were significant differences among groups of murderers by offender sex (i.e., female‐perpetrated sex worker homicides vs. sexual homicides, and male‐perpetrated sex worker homicides vs. sexual homicides). Relative to female sex worker homicide offenders, female SHOs were significantly more likely to be Whites (44% vs. 26%) and young adults (55% vs. 43%); and their victims to be Nonwhites (61% vs. 41%) and non‐strangers (81% vs. 65%). Female sex killers were also significantly more likely than female sex worker murderers to commit their offense in a lower populated area (e.g., suburban and rural areas; 34% vs. 15%), and to use a firearm (66% vs. 40%) and overall, a physically less demanding weapon (e.g., edged weapon and firearm; 95% vs. 85%) as their murder weapon. Nonetheless, there was no comparative analysis on male‐perpetrated and female‐perpetrated homicides in this study.

The present study builds on the earlier work by aiming to identify subtypes of female SHOs based upon heterogenous offender, victim, and offense characteristics and by employing an advanced statistical classification technique. Although there have been studies on empirically‐derived typologies of male SHOs (e.g., Balemba, Beauregard, and Martineau 2014; Chopin and Beauregard 2023; Healey et al. 2016; Mjanes, Beauregard, and Martineau 2017) and female sex offenders (e.g., Hong et al. 2023; Miller, Turner, and Henderson 2009; Turner, Miller, and Henderson 2008; Wijkman, Bijleveld, and Hendriks 2011), no empirical attempts have been performed on female SHOs. Therefore, the aim of this study is to develop the first statistically‐derived typology of female single‐victim (SV) sexual murderers in the U.S., and to explore the potential characteristically differences in their offending pattern. In addition to female sex killers whose offending patterns and process can be characteristically different from their male counterparts (Chan, Heide, and Beauregard 2019), heterogeneity was also found within the female sex offender population such as differences in background characteristics, victim choice, and offense context (Sandler and Freeman 2007; Wijkman, Bijleveld, and Hendriks 2011). Hence, learning about the potential different offender profiles of female sexual murderers may not only contribute to understanding the etiology of female sexual homicide offending, but also to offer more insights to guide law enforcement agents and security professionals in their investigation. Two research questions have been proposed for this study: (1) Is there latent heterogeneity in the characteristics of female SV SHOs in the U.S.? and (2) If so, how many female SV SHO subtypes exist and what are their distinguishing features? To do this, an advanced statistical technique, latent class analysis (LCA), was employed to derive the subtypes of female sexual murderers.

## 2 Method

### 2.1 Data and Procedure

The data analyzed in this study was drawn from the Federal Bureau of Investigation's (FBI) Supplementary Homicide Reports (SHRs) that spanned over a 47‐year period (1976–2022). The SHR data were compiled by Jacob Kaplan (2023) that consisted of demographic characteristics of both the offenders and victims, as well as the characteristics of each homicidal incident reported to the FBI. The data comprise of homicide reports that are voluntarily submitted by local law enforcement agencies that serve cities of varying sizes, suburban areas, and rural areas across all 50 U.S. states and the District of Columbia. Although reporting is voluntary, participation by law enforcement agencies across the U.S. is over 90%. Despite the small percentage of homicides that go undetected or unreported, the SHR data are perhaps the best available data on murder arrests in the U.S. (Chan 2021; Fox and Fridel 2017); and more importantly, these are the best available data to study female SHOs in the U.S. (Chan and Frei 2013; Chan, Frei, and Myers 2013). Reported incidents are commonly used to estimate the number of homicides nationally; and thus, arguably, these data provide the best representative sample of homicides in the U.S.

The sample of this study were selected from the SHR offender data based on two inclusion criteria: (1) they were arrested for murder or nonnegligent homicide, defined by the FBI, “as the willful (nonnegligent) killing of one human being by another,” and the homicide circumstance was coded as “rape” or “other sexual offenses” (Kaplan 2023). Of note, the classification of murder or nonnegligent homicide is based solely on police investigation as opposed to the determination of a court, medical examiner, coroner, jury, or other judicial body. The prosecutor's office in each jurisdiction makes the charging decision with respect to intent, namely first‐degree murder, second‐degree murder, or manslaughter. The homicides recorded in the SHR data were classified as sexual (i.e., rape or other sexual offense) by the investigating law enforcement agencies and not by the authors of this study. Hence, the reliability of the classifications of specific murders as sexually‐related by police officers and the extent to which the criteria the investigating officers adopted to make these determinations are similar to criteria used by field experts is unknown (Chan 2015; Ressler, Burgess, and Douglas 1988).

During the 47‐year period (1976–2022) under review, a total 791,358 individuals were arrested for homicides, of which 557,066 (88.4% males and 11.6% females) were arrested in single‐offender murders (no co‐offenders were reported). Out of this total, only 6201 cases (0.78%) with pertinent offense‐related data were identified as sexual in nature. Cases with missing values on variables under examination were listwise excluded from the analysis. A final total of 3362 cases (54.2%; 3204 male SHOs [95.3%] and 158 female SHOs [4.7%]) were identified as single‐victim‐single‐offender (SVSO) cases, and the rest (*N* = 2839) involved either multiple offenders, multiple victims, or unknown offenders. In this study, 158 female SV SHOs cases were analyzed. Multiple‐victim sexual homicide cases were excluded as they only represented about 10% of the entire female SHO population in this dataset. Importantly, studies have demonstrated that, to a certain extent, single‐victim SHOs are behaviorally and operationally differed from serial SHOs (with multiple victims) in their offending patterns and process (Campos and Cusson 2007; Chan, Beauregard, and Myers 2015).

### 2.2 Measures

Eight variables were examined in this study: Two pertained to the offender characteristics (i.e., age and racial groups), another three pertained to the victim characteristics (i.e., sex, and age and racial groups), and the remaining three pertained to the offense characteristics (i.e., offender‐victim relationship, geographical urbanness level, and offender's murder weapon type). Specifically, the offenders were categorized according to their age group as juvenile (age 7–17 years), young adult (age 18–39 years), or middle‐aged and older adult (age 40 years and above); while the victims were categorized into 5 different age groups: child (age 0–12 years), adolescent (age 13–17 years), young adult (age 18–39 years), middle‐aged adult (age 40–59 years), and older adult (age 60 years and above). The offenders and victims were also categorized by racial group as Black, White, American Indian or Alaskan Native, or Asian. The victims were also identified as either male or female.

There were three offense variables included in this study. The offender‐victim relationship was grouped as either intimate partner (e.g., current or former husband/wife or boy/girlfriend), immediate family members (e.g., biological or step‐parents, siblings, offsprings, or in‐law), other family members, friend/neighbor/colleague, acquaintance/other known relationship, or stranger. The level of geographical urbanness of the offense location was coded by the FBI as either higher populated area (i.e., large and small cities with populations of at least 2500) or lower populated area (i.e., suburban and rural areas). The types of murder weapon used by the offenders were categorized into four categories, namely personal weapon (i.e., killing with hands or feet, strangulation, beating with bare hands, asphyxiation, drowning, and defenestration [the act of throwing someone out of a window]), contact weapon (i.e., blunt object; e.g., baseball bat, golf club, hammer, and chair), edged weapon (i.e., different types of knives or bladed tools; e.g., kitchen knife, sword, machete, axe, and pocket knife), firearm (e.g., handgun, shotgun, and rifle), and other weapons (e.g., drugs, explosives, fire, and poison). These variables were selected as previous studies on sexual homicide that examined the SHR data have found significant differences in both offender sex, male (e.g., Chan and Beauregard 2016; Chan and Heide 2008; Chan, Myers, and Heide 2010, 2013; Myers and Chan 2012; Myers et al. 2017) and female (e.g., Chan and Frei 2013; Chan, Frei, and Myers 2013, 2019) sexual murderers.

### 2.3 Data Analytic Strategy

An LCA was applied to classify female SV SHOs according to their offending dynamics (i.e., offender, victim, and offense characteristics). According to Nylund, Asparouhov, and Muthén (2007), LCA models are used to “uncover unobserved heterogeneity in a population and to find substantively meaningful groups of people that are similar in their responses to measured variables” (p. 536). LCA is performed to determine whether unique latent classes of cases exist in the data through the analysis of categorical variables (Weller, Bowen, and Faubert 2020). Simply put, LCA relies on probabilistic assignment to classes based on similar response on item response profiles, which is relevant in this study given the nature of the data. This advanced statistical classification technique is a “person‐focused” analytical method with its popularity has grown in recent years in classifying offending populations that are heterogenous in their characteristics (Francis, Bowater, and Soothill 2004). Relevant to this study, LCA has been used to identify distinct offending patterns of violent sex offenders (e.g., Deslauriers‐Varin and Beauregard 2010; Healey et al. 2016), and more relatedly, on homicide offenders using the SHR dataset (e.g., Cochran et al. 2024; Michel et al. 2024).

LCA was selected over other classification techniques and factor analysis because it relaxes the assumptions of normal distribution, linearity, and homogeneity of variance. This statistical approach is also suitable for small datasets, such as the present study, and those that cannot be analyzed using conventional parametric clustering techniques (Magidson and Vermunt 2004; Sinha, Calfee, and Delucchi 2021). Further, this classification technique allows small sample sizes with simpler models (having fewer indicators and classes) and “well‐separated classes” (Weller, Bowen, and Faubert 2020). Nylund‐Gibson and Choi (2018) posited that for simple LCA models with a pair of well‐separated classes, a sample size as small as 30 may be sufficient. In this study, the SnowRMM module in Jamovi 2.3.28 version program was used for data analysis, and the LCA was performed in sequence, beginning from the 2‐class model (the default) to the 7‐class model (see Table 1) (The Jamovi Project 2024). The models were compared using statistical and substantive theoretical criteria to determine the best model. The optimal number of class membership was determined by a combination of goodness‐of‐fit criteria: Bayesian Information Criterion (BIC), Consistent Akaike Information Criterion (CAIC), likelihood‐ratio statistic (G^2^), and entropy value (0.0–1.0) (Garthe, Sullivan, and Behrhorst 2021). The BIC, calculated based on the sample size and the number of parameters, is gradually being credited as the most trustworthy (Nylund, Asparouhov, and Muthén 2007; Weller, Bowen, and Faubert 2020) and the most widely reported in LCA studies (Killian et al. 2019). A lower BIC value is desirable.

**TABLE 1:** Fit indices for all potential class solutions using LCA.

No. of classes	G^2^	AIC	BIC	CAIC	LL	Entropy	*χ* ^2^
2	571	2011	2149	2194	−960	0.977	23,007
3	522	2066	2214	2282	−935	0.887	20,780
4	486	2016	2295	2386	−917	0.871	24,437
5	428	2005	2354	2468	−889	0.910	9381
6	429	2053	2473	2610	−890	0.920	4091
7	372	2037	2527	2687	−858	0.913	6273

Abbreviations: AIC, Akaike information criterion; BIC, Bayesian information criterion; CAIC, consistent AIC; G^2^, likelihood ratio statistics; LL, log‐likelihood; *χ* ^2^, Pearson chi‐square goodness of fit statistic.

## 3 Results

### 3.1 Descriptive Statistics

The offender, victim, and offense characteristics of 158 female SV SHOs are shown in Table 2. Female SHOs averagely aged 27.96 years (*SD* = 10.42), with three quarters of them (75.3%) were young adults (18–39 years). Female offenders in this study were mostly Black (53.2%) and White (44.3%) in their racial composition. The murder they committed was heterogenous in nature, with mostly male victims (88.6%). Half of their victims (52.6%) were young adults (18–39 years) and another 31.4% were middle‐aged adults (40–59 years); with a mean age of 38.28 years (*SD* = 15.96). Nearly all victims in this study were either Black (58.6%) or White (40.1%) in their racial background. Concerning the offender‐victim relationship, female SHOs tended to target those whom they had lesser close relationship, being 42% were acquaintances (or with other known relationship) and 33.9% were strangers. These offenders were more likely to commit their murder in higher populated areas (76.6%), with firearms being the most commonly used weapon (63.9%) in their murder.

**TABLE 2:** Frequencies of offender, victim, and offense characteristics of female single‐victim sexual homicides sample extracted from the *Uniform Crime Reports [United States]*: *Supplementary Homicide Reports*, *1976–2022* (*N* = 158).

Characteristics	*n*	Percentage
Offender characteristics		
Offender mean age	27.96 (*SD* = 10.42)	Range = 14–76
Offender age group	(*N* = 158)	
Juvenile (7–17 years)	17	10.8%
Young adult (18–39 years)	119	75.3%
Middle‐aged and older adult (40 years and above)	22	13.9%
Offender racial group	(*n* = 157)	
Black	80	53.2%
White	70	44.3%
American Indian, Alaskan Native	3	1.9%
Asian	1	0.6%
Victim characteristics		
Victim mean age	38.28 (*SD* = 15.96)	Range = 1–90
Victim age group	(*n* = 156)	
Child (0–12 years)	5	3.2%
Adolescent (13–17 years)	3	1.9%
Young adult (18–39 years)	82	52.6%
Middle‐age adult (40–59 years)	49	31.4%
Older adult (60 years and above)	17	10.9%
Victim sex	(*N* = 158)	
Female	18	11.4%
Male	140	88.6%
Victim racial group	(*n* = 157)	
Black	92	58.6%
White	63	40.1%
American Indian, Alaskan Native	2	1.3%
Offense characteristics		
Offender‐victim relationship	(*n* = 151)	
Intimate partner	29	5.4%
Immediate family member	19	3.3%
Other family members	5	3.2%
Friend/neighbor/colleague	10	12.2%
Acquaintance/other known	62	42.0%
Stranger	26	33.9%
Geographical urbanness level	(*N* = 158)	
Higher populated areas	121	76.6%
Lower populated areas	37	23.4%
Murder weapon type	(*N* = 158)	
Personal weapon	10	6.3%
Contact weapon	6	3.8%
Edged weapon	40	25.3%
Firearm	101	63.9%
Other weapons	1	0.6%

### 3.2 2| Identifying Latent Subgroups of Female SV SHOs

There is no definitive statistical value for determining the optimal number of classes to select. Oftentimes, the model with the lowest values is selected as these measures indicate a better fit to the data than models with higher values (Keribin 2000). Lower statistical values typically produce more parsimonious models. However, it is not often that a model will generate the minimum values for all criteria measures, so the model with the majority of measures in the lowest value will normally be selected. From the model fit statistics, a 2‐class model was found to provide the best solution to the data (see Table 1).

As the final evaluation of model fit, the 2‐class model was externally validated using chi‐square analyses with the Bonferroni correction method (comparing the column proportions) to determine if the majority of variables in different classes distinguish from one another to be distinctive groups (Keller, Cusick, and Courtney 2007). Findings of the validation analyses, in Table 3, indicate that six out of eight variables (with the exceptions of the offender age group and victim sex) were statistically significant at the *p* < 0.05 level. Post‐hoc tests was subsequently computed and indicated that the two offender groups differed significantly.

**TABLE 3:** Latent classes of female single‐victim sexual homicide offenders (*N* = 158).

Variables	Class 1	Class 2	Differences		
	White intra‐familial offenders	Black extra‐familial offenders	*χ* ^2^ (df)	Phi/Cramer's	*p* value
Class prevalence	39.6% (63)	60.4% (95)			
Offender age group			4.23 (2)	0.16	*p* > 0.05
Juvenile	14.3%	8.4%			
Young adult	66.7%^a^	81.1%^a^			
Middle‐aged and older adult	19.0%	10.5%			
Offender racial group			119.47 (3)	0.87	*p* < 0.001
Black	0.0%	88.4%			
White	95.2%^b^	10.5%^b^			
American Indian, Alaskan Native	3.2%	1.1%			
Asian	1.6%	0.0%			
Victim age group			14.29 (4)	0.30	*p* = 0.006
Child	3.2%	3.2%			
Adolescent	0.0%	3.2%			
Young adult	41.3%^c^	60.2%^c^			
Middle‐age adult	47.6%^d^	20.4%^d^			
Older adult	7.9%	12.9%			
Victim sex			0.01 (1)	−0.01	*p* > 0.05
Female	11.1%	11.6%			
Male	88.9%	88.4%			
Victim racial group			145.04 (2)	0.96	*p* < 0.001
Black	0.0%	96.8%			
White	96.8%^e^	3.2%^e^			
American Indian, Alaskan Native	3.2%	0.0%			
Offender‐victim relationship			17.82 (5)	0.34	*p* = 0.003
Intimate partner	25.4%	14.8%			
Immediate family member	22.2%^f^	5.7%^f^			
Other family members	4.8%	2.3%			
Friend/neighbor/colleague	6.3%	6.8%			
Acquaintance/other known	33.3%	46.6%			
Stranger	7.9%^g^	23.9%^g^			
Geographical urbanness level			25.83 (1)	−0.40	*p* < 0.001
Higher populated areas	55.6%^h^	90.5%^h^			
Lower populated areas	44.4%^i^	9.5%^i^			
Murder weapon type			9.67 (4)	0.25	*p* = 0.046
Personal weapon	3.2%	8.4%			
Contact weapon	6.3%	2.1%			
Edged weapon	15.9%^j^	31.6%^j^			
Firearm	74.6%^k^	56.8%^k^			
Other weapons	0.0%	1.1%			

*Note:* Subscripts (a, b, c, d, e, f, g, h, i, j, k) indicate significant differences.

The first class accounted for 39.6% of the sample. Female SV SHOs in this class included predominantly White offenders (95.2%) with their victim mainly middle‐aged (47.6%) and young adults (41.3%). The murder they committed was largely intra‐racial (i.e., White killed White; 96.8%) and intra‐familial (e.g., intimate partner, immediate family member, and other family members; 52.4%) in nature. Offenders in this class were more preferred to use firearm (74.6%) as their murder weapon. This class was labeled as *White intra‐familial offenders*.

The second class accounted for 60.4% of the sample. Female SV SHOs in this class included predominantly Black (88.4%) young adults (81.1%), and were more likely to murder victim in the same age group—that is, young adults (60.2%). Similarly, offenders in this class tended to murder intra‐racially (i.e., Black killed Black; 96.8%), but the offense committed was mostly inter‐familial in nature (e.g., stranger, acquaintance, and other with known relationship; 70.5%). Although a firearm (56.8%) was most commonly used among these offenders, they were also more likely to use an edged weapon (31.6%) in killing their victim. Their murder was predominantly committed in a higher populated area (90.5%). This class was labeled as *Black inter‐familial offenders*.

## 4 Discussion

Analyzing the FBI's SHR dataset that spanned over a 47‐year period (1976–2022), the present study aimed to classify sexual homicides perpetrated by female offenders. This study explored different offender, victim, and offense characteristics using LCA to analyze a sample of 158 female SV SHOs. To the best of the authors' knowledge, this study is the first to empirically classify female SHOs. While the findings may not generalize to female sexual murders in other countries and also those who murdered more than one victim, the present study provides an initial step in classifying typology with an under‐researched population of female SHOs. The offender classification found in this study may inform police practice in the area of suspect prioritization and help police strategize their investigation efforts. Besides, this offender classification may be informative for behavioral scientists and mental health professionals (e.g., forensic psychologists, psychiatrists, and criminal and mental health attorneys) who advise law enforcement officers in the investigation of such offenses. The use of LCA to identify the latent profile of female SV sexual murderers suggests that two distinct groups of female offenders.

### 4.1 Class 1: White Intra‐Familial Female SHOs (40%)

This class of female offenders is predominantly Whites whose victims are from the same racial background—that is, White victims. The intra‐racial killing by White sexual murderers is a frequently found offending pattern across both male and female offenders (e.g., Chan, Myers, and Heide 2010; Skott, Beauregard, and Darjee 2019). Offenders in this class are more likely to select middle‐aged adults as victims but also somewhat likely to targets young adults. This victim age trend in target selection is relatively in line with female murderers in general (e.g., Farrell, Keppel, and Titterington 2013; Heide et al. 2012). Consistent with the finding by Koons‐Witt and Schram (2006) where White female murderers are more likely to target intimate partners, the defining feature of this offender class is that White female SHOs are more likely to select victims within their family network—that is, intimate partner (e.g., former or current spouse, boy/girlfriend, and sexual partner) and family member (e.g., biological or step‐parents, siblings, offsprings, and in‐laws). Although the murder is more likely to occur in a higher populated area (e.g., a large and small city with a population of at least 2500), it is also somewhat likely to occur in a lower populated area (e.g., a suburban and rural area).

Another distinguishing feature of this class is that the offenders are more likely to use a firearm (e.g., handgun, shotgun, and rifle) in killing their victim. Relative to male SHOs, female SHOs in general are more likely to prefer the use of firearms as their murder weapon (Chan, Heide, and Beauregard 2019). Chan and Beauregard (2016) posited that the type of weapon used is likely to be influenced by the practicality of weapon choice. The physical strength hypothesis in sexual homicides suggests that the offenders who are physically weaker and less capable of overcoming their victims' resistance (e.g., female‐on‐male sexual homicide) are more likely to select weapons that require fewer physical strength (e.g., firearms) (Chan and Heide 2008). In this study where the victims were predominantly males who were likely to be physically stronger than the female offenders, a murder weapon that required lesser physical strength may be preferred.

### 4.2 Class 2: Black Extra‐Familial Female SHOs (60%)

The second class of female SHOs also suggests an intra‐racial kind of female sexual homicides, with Black offenders are more likely to sexually murder Black victims. Young adults are more likely to be targeted by this class of female offenders. The murder committed by this class of offenders is most likely to occur in a higher populated area. Consistent with previous study on female Black sexual murderers (Chan, Frei, and Myers 2013), this class of female SHOs are more likely to target victims outside of their family network—for example, stranger and acquaintance. Although the offenders are more likely to use a firearm to kill their victim, they are also somewhat likely to use an edged weapon (i.e., different types of knives or bladed tools; e.g., kitchen knife, sword, machete, axe, and pocket knife) as their murder weapon. Compared to types of murder weapons, firearms and edged weapons are found to be more frequently used by female murderers in general (e.g., Chan, Heide, and Beauregard 2019; Heide et al. 2011 2012). Aside from firearms, edged weapons are also considered as a less physically demanding murder weapon. Unlike male sexual murderers who primarily engaged in an intimate or close‐contact killing (e.g., manual or ligature strangulation, and asphyxiation) that can provide more psychological excitement or sexual euphoria (Chan and Beauregard 2016; Chan and Li 2020), female sexual murderers who used an edged weapon or a firearm can commit a quick and easy kill. Such killing methods inherently prevent strong physical resistance by the victim due to minimal physical contact.

## 5 Conclusion

In general, this study provides an important initial glimpse into the different subpopulations of female SHOs that have occurred in the U.S. over a period that spanned 47 years (1976–2022). However, the findings of this study should be interpreted cautiously in view of several limitations. First, the SHR dataset was compiled from arrest records and not convictions. Thus, it remains unclear if these arrested individuals were subsequently charged and/or convicted. Second, the SHR dataset is limited to basic offender, victim, and offense circumstance variables. Such level of detail does not allow for more in‐depth examination into offense‐related factors (e.g., victim‐offender interactions and situational characteristics), offender motivation, psychopathology, and comprehensive case characteristics that are regularly collected in clinical and correctional settings (DeLisi et al. 2023; Peters, Bonner, and DeLisi 2024). Third, the findings in this study are limited to known cases. It is possible that offenders who avoided police detection and evaded arrest, and thus have a dark figure of murder (Minkler et al. 2024) have different offending patterns. Fourth, these cases were coded by the participating law enforcement agencies; and hence, reporting errors, misclassifications, or omissions is likely to occur. Among these errors, missing data are by far the most problematic issue with the SHR data (Fridel and Fox 2019). Data from individual cases may record as missing when participating agencies fail to report some or all of their homicide cases to the FBI. Fifth, concerning the analysis, small category size in some variables may bias the estimates that were produced.

Notwithstanding the limitations, this study is important in not only advancing our knowledge of sexual homicide from an under‐researched population, but more importantly, to propose an initial empirically‐derived offender classification of female SHOs. As such, this study has established a solid foundation for further research to better comprehend the offending dynamics of female sexual homicides. It is utterly important to continue building our knowledge about this rarely examined offender population in which implications for practice (e.g., investigative strategy) could be more effectively and efficiently proposed. For instance, if the White young or middle‐aged male victim was murdered by a firearm and the murder was ruled sexual in nature, the police may have a reason to believe that the offender was a White female adult within the family network (i.e., intra‐familial homicide). Alternatively, if the Black young male victim was killed by either a firearm or an edged object (e.g., knife) in a city and the murder was regarded as sexually‐motivated, the police may have a reason to suspect that the offender was a Black young female adult who was not family‐related (i.e., extra‐familial homicide).

Nevertheless, cautious interpretation is necessary given the study's small sample size and the correlational nature of the analysis. Multiple factors (e.g., victim‐offender interactions and situational circumstances) may influence the decision‐making of the offenders prior or during the offense and ultimately the outcome of the offense, which was limited by the dataset examined in this study. Therefore, future empirical research on female SHOs should carefully consider the depth of information required to obtain a more comprehensive understanding of this unique offender population. Identifying the underlying causal or predisposing factors (e.g., the offender's developmental vulnerability, motives, and psychopathology) and other offending behavior (e.g., post‐crime behavior) may not only advance the forensic field's knowledge about female SHOs, but also to better assist law enforcement agents and security professionals in their investigative strategies. For instance, this could inform police practice in the form of suspect prioritization (i.e., offender profiling) in such a way to better strategize the police investigation plans.

## Ethics Statement

The authors have nothing to report.

## Conflicts of Interest

The authors declare no conflicts of interest.
